# Supplementary material for: Broad-Spectrum Efficacy and Modes of Action of Two Bacillus Strains against Grapevine Black Rot and Downy Mildew
Source: J Fungi (Basel). 2024 Jul 9;10(7):471. doi: 10.3390/jof10070471 (PMC11278100; doi:10.3390/jof10070471)
Supplement: Supplementary file 1 [file jof-10-00471-s001.zip › Supplementary Table S1 - List of primers used for the assessment of defense-related responses.pdf]

Name		Forward Primer (5’ -> 3’)	Reverse Primer (5’ -> 3’)
**VATP16**	V-type proton ATPase 16kDa subunit	CTTCTCCTGTATGGGAGCTG	CCATAACAACTGGTACAATCGAC
**EF1α**	Elongation Factor 1 - alpha	TCTGCCTTCTTCCTTGGGTA	GCACCTCGATCAAAAGAGGA
**STS1.2**	Stilbene synthase	AGGAAGCAGCATTGAAGGCTC	TGCACCAGGCATTTCTACACC
**ROMT**	Resveratrol O-methyltransferase	TGCCTCTAGGCTCCTTCTAA	TTTGAAACCAAGCACTCAGA
