# Supplementary material for: Broad-Spectrum Efficacy and Modes of Action of Two Bacillus Strains against Grapevine Black Rot and Downy Mildew
Source: J Fungi (Basel). 2024 Jul 9;10(7):471. doi: 10.3390/jof10070471 (PMC11278100; doi:10.3390/jof10070471)
Supplement: Supplementary file 1 [file jof-10-00471-s001.zip › Supplementary Table S2 - Quantification of the different studied stilbenes.pdf]

**Stilbenes**	**Quantified as**
trans-resveratrol	
cis-piceid	as *trans*-piceid
trans-piceid	
piceid isomer	as *trans*-piceid
trans-piceatannol	
atringin isomer 1	as *trans*-astringin
astringin isomer 2	as *trans*-astringin
piceatannol isomer	as *trans*-piceatannol
isorhapontin	
pterostilbene	
trans-δ-viniferin	
trans-ε-viniferin	
viniferin isomer 1	as *trans*-ε-viniferin
viniferin isomer 2	as *trans*-ε-viniferin
trans-ω-viniferin	
