# Supplementary figures and images for: Broad-Spectrum Efficacy and Modes of Action of Two Bacillus Strains against Grapevine Black Rot and Downy Mildew
Source: J Fungi (Basel). 2024 Jul 9;10(7):471. doi: 10.3390/jof10070471 (PMC11278100; doi:10.3390/jof10070471)

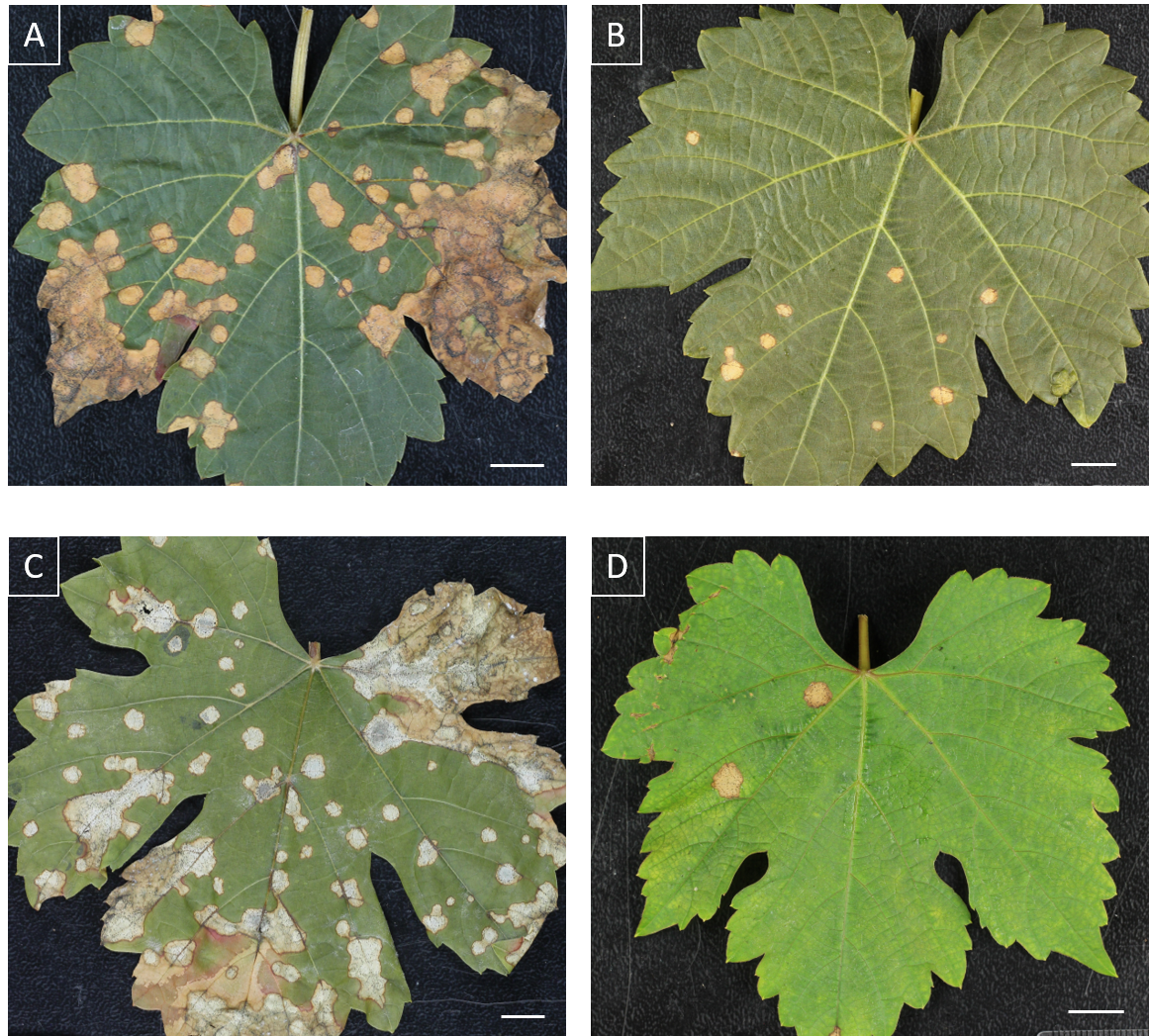

Supplement: Supplementary file 1 [file jof-10-00471-s001.zip › Supplementary Figure S1 - Black rot typical lesions on Artaban (A and B) and Marselan grapevine varieties, corresponding to high (A and C) and low (B and D) severity levels..png]

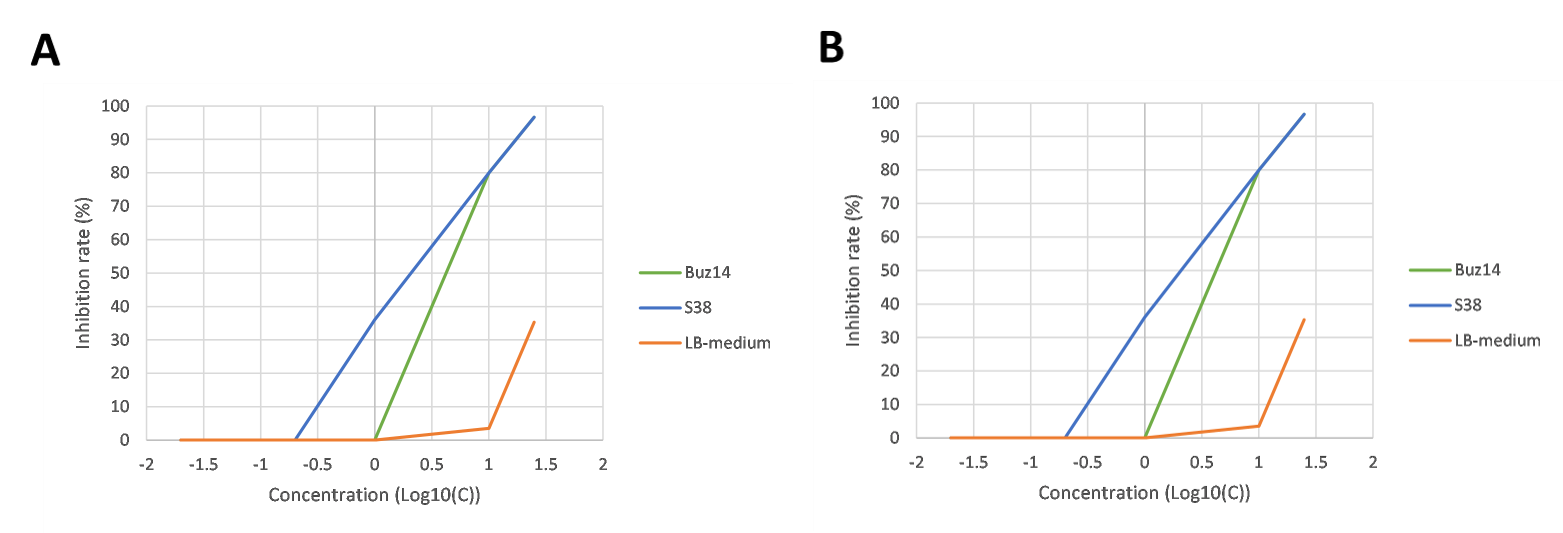

Supplement: Supplementary file 1 [file jof-10-00471-s001.zip › Supplementary Figure S2 - G. bidwelli fungal growth inhibition dose response curves, for Vitis specific strain (A) and Parthenocissus specific strain (B).png]
